# The effects of altered DNA damage repair genes on mutational processes and immune cell infiltration in esophageal squamous cell carcinoma

**DOI:** 10.1002/cam4.5663

**Published:** 2023-01-27

**Authors:** Huangbo Yuan, Tao Qing, Sibo Zhu, Xiaorong Yang, Weicheng Wu, Kelin Xu, Hui Chen, Yanfeng Jiang, Chengkai Zhu, Ziyu Yuan, Tiejun Zhang, Li Jin, Chen Suo, Ming Lu, Xingdong Chen, Weimin Ye

**Affiliations:** ^1^ State Key Laboratory of Genetic Engineering, Human Phenome Institute, and School of Life Sciences Fudan University Shanghai China; ^2^ Breast Medical Oncology, School of Medicine Yale University Connecticut New Haven USA; ^3^ Clinical Epidemiology Unit Qilu Hospital of Shandong University Jinan China; ^4^ Department of Biostatistics, School of Public Health Fudan University Shanghai China; ^5^ Fudan University Taizhou Institute of Health Sciences Taizhou China; ^6^ Department of Epidemiology, School of Public Health Fudan University Shanghai China; ^7^ National Clinical Research Center for Aging and Medicine, Huashan Hospital Fudan University Shanghai China; ^8^ Yiwu Research Institute of Fudan University Yiwu China; ^9^ Department of Medical Epidemiology and Biostatistics Karolinska Institute Stockholm Sweden

**Keywords:** DNA damage repair, esophageal squamous cell carcinoma, immune cell infiltration, next‐generation sequencing

## Abstract

**Background:**

Defects in DNA damage repair (DDR) pathways lead to genomic instability and oncogenesis. DDR deficiency is prevalent in esophageal squamous cell carcinoma (ESCC), but the effects of DDR alterations on mutational processes and tumor immune microenvironment in ECSS remain unclear.

**Methods:**

Whole‐exome and transcriptome sequencing data of 45 ESCC samples from Taizhou, China, were used to identify genomic variations, gene expression modulation in DDR pathways, and the abundance of tumor‐infiltrating immune cells. Ninety‐six ESCC cases from The Cancer Genome Atlas (TCGA) project were used for validation.

**Results:**

A total of 57.8% (26/45) of the cases in the Taizhou data and 70.8% (68/96) of the cases in the TCGA data carried at least one functional impact DDR mutation. Mutations in the DDR pathways were associated with a high tumor mutation burden. Several DDR deficiency‐related mutational signatures were discovered and were associated with immune cell infiltration, including T cells, monocytes, dendritic cells, and mast cells. The expression levels of two DDR genes, *HFM1* and *NEIL1*, were downregulated in ESCC tumor tissues and had an independent effect on the infiltration of mast cells. In the Taizhou data, increased expression of *HFM1* was associated with a poor prognosis, and the increased expression of *NEIL1* was associated with a good outcome, but no reproducible correlation was observed in the TCGA data.

**Conclusion:**

This research demonstrated that DDR alterations could impact mutational processes and immune cell infiltration in ESCC. The suppression of *HFM1* and *NEIL1* could play a crucial role in ESCC progression and may also serve as prognostic markers.

## Graphical abstract

DNA damage repair (DDR) deficiency leads to genomic instability and oncogenesis and is prevalent in esophageal squamous cell carcinoma (ESCC). Using whole‐exome and transcriptome sequencing data from Taizhou and TCGA, we demonstrated that DDR alterations could impact mutational processes and immune cell infiltration in ESCC. The suppression of HFM1 and NEIL1 could play a crucial role in ESCC progression and may also serve as prognostic markers.

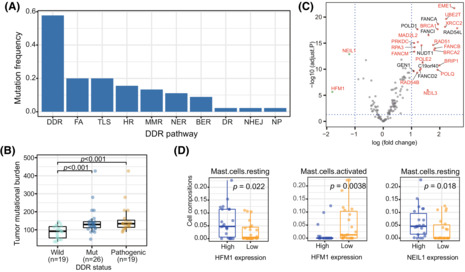

## 1 INTRODUCTION

Esophageal carcinoma (EC) is the seventh most common and the sixth most lethal cancer worldwide.^1^ Esophageal squamous cell carcinoma (ESCC), the predominant histological subtype within EC, is a highly progressive malignant tumor with a 5‐year overall survival rate of less than 20% for patients with advanced tumor stages.^2^ Although immunotherapy has become a promising ESCC treatment according to recent clinical trials, the response rate is relatively low among unselected patients.^3^, ^4^ Therefore, it is essential to find reliable prognostic biomarkers and targetable molecular alterations of ESCC to develop more effective treatments.

DNA damage repair (DDR) pathways have crucial roles in maintaining genomic integrity under normal cellular conditions.^5^ DDR deficiency can result in the accumulation of somatic mutations, which could produce additional changes in cell physiology that drive tumor initiation.^6^ Genomic alterations of DDR pathways are prevalent across various cancer types.^7^ A previous study reported that DDR mutations were detected in esophageal squamous dysplasia tissues and were more common in ESCC tissues.^8^ Therefore, failure of DDR is one of the critical drivers for the tumorigenesis of ESCC. Although the association between DDR deficiency and tumor mutation burden (TMB) has been well explored in many cancers, the underlying mechanisms involved in tumorigenesis caused by DDR deficiency in ESCC remain much less explored.^9^, ^10^, ^11^

In addition to playing a role during tumorigenesis, DDR deficiency is vital in driving the antitumor immune response. Tumor cells with DDR deficiency tend to have a high TMB, which is believed to be a key driver in the generation of immunogenic neoantigens displayed on the major histocompatibility complexes (MHC) on the tumor cell surface that influence patient response to immunotherapy.^7^, ^12^ A high TMB and mutations in some DDR pathways, such as mismatch repair deficiency (dMMR), have potential for predicting the efficacy of immune checkpoint blockade (ICB).^13^

Nevertheless, only a few of the mutations in cancers that are expected to be immunogenic are recognized by T cells. Thus, only a small number of patients identified by these biomarkers can benefit from ICB.^14^ Therefore, exploring the genomic determinants of the tumor immune environment is essential.

Several DDR pathways have been implicated in the infiltration of immune cells in some solid tumors, but limited data exist detailing the impact of DDR deficiency on immune cell infiltration in ESCC.^9^, ^15^, ^16^ Understanding this impact may provide insight into the development of ESCC and yield a promising biomarker for immunotherapy response.

In the present study, we investigated the genomic and transcriptomic alterations of 160 DDR genes from nine DDR pathways in 45 Taizhou ESCC cases and 96 TCGA ESCC cases. We identified ubiquitous deficiencies in the DDR in ESCC and showed their essential role in mutational processes. We then evaluated the profiles of immune cell infiltration utilizing the CIBERSORT algorithm and confirmed the association between mutations and immune cell infiltration. Finally, we discovered the suppression of *HFM1* and *NEIL1* and revealed their effects on mast cells and patient outcomes. These findings highlight the importance of the DDR in the tumorigenesis of ESCC and show its potential applications for predicting the response to tumor immunotherapy.

## 2 MATERIALS AND METHODS

### 2.1 Sample collection

Tumors and matched normal tissues were obtained from patients who had been diagnosed with ESCC at Taixing People's Hospital in Taizhou city in China. Tissues were collected during surgery and immediately stored at −80°C. All samples were collected from ESCC patients who received surgery as their primary treatment from 2014 to 2017 at Taixing People's Hospital. Two pathologists checked the samples independently to confirm the tumor type and purity. Finally, 45 tumors (composition >50% tumor cells) and matched normal tissues were selected for sequencing. The clinical features of the patients are summarized in Table 1.

**TABLE 1:** Clinical features of ESCC patients in this study.

Clinical features	Taizhou ESCC *N* = 45	TCGA ESCC *N* = 96
Age, median [range], years	69 [50–82]	57 [36–90]
Gender, *n* (%)		
Male	34 (75.6%)	81 (84.4%)
Female	11 (24.4%)	15 (15.6%)
Race, *n* (%)		
Asian	45 (100%)	45 (46.9%)
White	0	43 (44.8%)
Black or African American	0	5 (5.2%)
Missing	0	3 (3.1%)
Tumor Stage, *n* (%)		
I	12 (26.7%)	7 (7.3%)
II	14 (31.1%)	56 (58.3%)
III	19 (42.2%)	27 (28.1%)
IV	0	4 (4.2%)
Missing	0	2 (2.1%)
Survival, *n* (%)		
Dead	21 (51.1%)	32 (33.3%)
Alive	17 (35.6%)	64 (66.7%)
Unknown	7 (13.3%)	0

### 2.2 Next‐generation sequencing

Using the exome capture procedure, DNA was extracted from the collected samples using SeqCap EZ Human Exome Probes v3.0 kits (Roche Sequencing Solutions, Inc.). The captured DNA samples were sequenced using the HiSeq X Ten system. The paired‐end 150 bp sequencing mode was employed to yield an average of 139.8 million high‐quality reads of 21.0 Gb bases for each of the 90 samples.

Total RNA (>18 nucleotides) was extracted from the tissues using the miRNeasy Mini Kit with DNase treatment (Qiagen). All RNA samples had the RNA integrity numbers (RINs) > 7.5. Libraries for tumor and normal tissues were prepared using Ribo‐Zero rRNA Removal Kit coupled with the Illumina RNA‐sequencing (RNA‐seq) library protocol. One of the tumor RNA libraries failed to pass our quality control due to RNA degradation (concentration = 3.9 ng/μL, A260/230 value = 0.07). The remaining 89 RNA samples were sequenced using the Illumina HiSeq 2500 platform.

### 2.3 Exome sequencing data analysis

The high‐quality reads were mapped to the human reference genome (GRCh38) using Burrows–Wheeler Aligner (BWA v0.7.17).^17^ The duplicated sequences and alignment adjustment were analyzed using GATK (v4.2.4).^18^ Mutect2 (v4.1) and Strelka2 (v2.9.2) were used to analyze the somatic substitutions and indels.^19^, ^20^ Somatic mutations had to be identified by both methods, and then were annotated using the Ensembl Variant Effect Predictor (VEP, v102.0).^21^ Finally, we detected 12,509 single nucleotide variants (SNVs) and 466 insertion–deletion mutations (indels) across all samples.

The tumor mutation burden (TMB) was defined as the number of mutations (excluding synonymous mutations) in the tumor tissue. The mutational signatures were extracted using the R package *sigminer* (v2.1.4) based on nonnegative matrix factorization (NMF) with default options.^22^ Briefly, the best number of decomposition modules was selected and subsequently used to match the signatures to previously identified COSMIC signatures.^23^

According to the literatures, a set of 160 DDR genes from 9 DDR pathways was summarized (see Table S1), including base excision repair (BER), direct reversal of damage (DR), mismatch repair (MMR), nucleotide excision repair (NER), homologous recombination (HR), Fanconi anemia (FA), nonhomologous end joining (NHEJ), modulation of nucleotide pools (NP), and translesion DNA synthesis (TLS).^24^, ^25^, ^26^ We considered four mutation classifications in our following analysis, including missense mutation, frameshift insertion, frameshift deletion, and nonsense mutation, which might impact the function or expression of a gene. The mutation frequency for each DDR pathway was estimated as the percentage of individuals carrying at least one functional impact mutation in any of the DDR genes involved. We separated the ESCC samples into DDR‐mutated and DDR‐wild‐type groups based on the mutation status of these 160 DDR genes. Considering the heterogeneous of missense mutations, we classified missense mutations as pathogenic and benign mutations and repeated the same analysis after excluding the benign mutations to see the stability of the results. Briefly, any missense mutation annotated as “HIGH” in the VEP annotated column IMPACT, “deleterious” in the column SIFT, “damaging” in the column PolyPhen or INDELs/nonsense mutation in the coding region of DDR genes was defined as a pathogenic mutation. Any missense mutation annotated as “tolerated” in the column SIFT, “benign” in the column PolyPhen or “LOW/ MODERATE” in the column IMPACT was defined as a benign mutation.

### 2.4 RNA‐seq data analysis

The raw sequencing reads were processed with Trimmomatic (v0.35) to remove the sequencing adapter and the low‐quality data.^27^ The high‐quality RNA‐seq reads were mapped to the human reference genome (GRCh38) using HiSat2 (v2.0.5) with default parameters.^28^ The featureCounts algorithm was used for the quantification of RefSeq genes (hg38).^29^, ^30^ The raw counts of each gene were normalized to the reads per million sequenced reads, and then log2 transformed. The differentially expressed genes (DEGs) were identified by comparing the gene expression between the tumor and normal samples using the *limma* R package.^31^ We used log fold change (logFC) > 1 or <−1 and a false discovery rate (FDR)‐adjusted *p* value < 0.05 as criteria to select the differentially expressed genes. The R packages *clusterProfiler* and *topGO* were used to perform enrichment analysis of gene ontology (GO) and Kyoto Encyclopedia of Genes and Genomes (KEGG) pathway.^32^, ^33^

### 2.5 Public dataset analysis

The somatic mutations and relevant clinical data of the additional 96 ESCC samples were obtained from The Cancer Genome Atlas (TCGA) project. The clinical features of the patients are summarized in Table 1. The normalized RNA‐seq data of 95 ESCC tumor tissues and 321 normal esophageal tissues from TCGA and the Genotype‐Tissue Expression (GTEx) projects were downloaded using the R package *TCGAbiolinks*.^34^ The downloaded RNA‐seq data were from the ReCount2 project (https://jhubiostatistics.shinyapps.io/recount/) that processes and unifies raw sequencing data from both platforms after uniform realignment, gene expression quantification, and batch effect removal.^35^

### 2.6 Estimation of immune landscapes

CIBERSORT (https://cibersort.stanford.edu/) and leucocyte signature matrix 22 (LM22) were used to quantify the relative fraction of 22 immune‐cell types in the samples.^36^ Normalized gene expression data were analyzed using the CIBERSORT algorithm with 1000 permutations. The CIBERSORT *p* value reflects the statistical significance of the results, and a threshold less than 0.05 is recommended. Finally, samples with CIBERSORT *p* values less than 0.05 were included in the following analyses.

### 2.7 Statistical analysis

All statistical tests and graphing were performed with the R program (v4.1.3). Clinical variables were summarized using percentages and descriptive statistics (mean, range, frequencies). We estimated the correlation between two quantitative data with Pearson's correlation analysis. The Kruskal–Wallis test was performed to evaluate the difference between groups. Linear regression analysis was conducted to identify the correlation between the DDR gene expression and the proportions of infiltrating immune cells. Differences in overall survival (OS) were assessed with Kaplan–Meier curves, and statistical significance was calculated using the log‐rank test. The thresholds for the different prognostic risk groups were calculated using the R package *maxstat*.^37^ Hazard ratios (HRs) and associated 95% confidence intervals (CIs) were determined by Cox regression. Each *p* value was two‐sided, and a *p* value < 0.05 was considered statistically significant. The *p* values of multiple tests were corrected by the FDR approach.

## 3 RESULTS

### 3.1 Genomic alterations of DDR genes in ESCC

**FIGURE 1:**
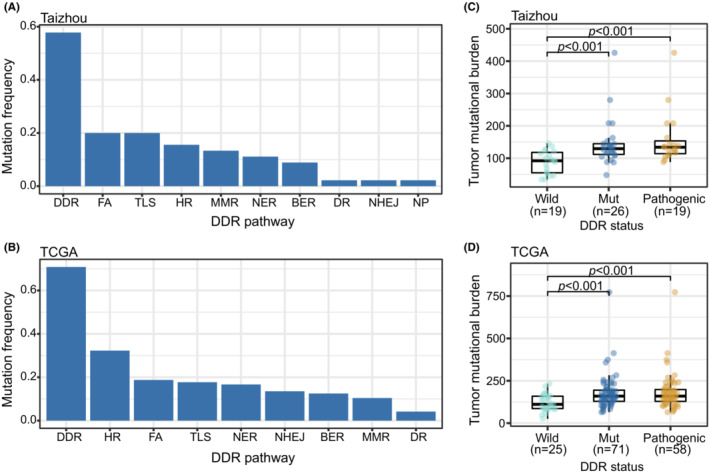
Genomic alterations of DNA damage repair (DDR) genes. (A, B) Mutation frequencies of DDR pathways in the Taizhou data (A) and TCGA data (B). (C, D) Tumor mutation burdens (TMB) of DDR groups in the Taizhou data (C) and TCGA data (D). TMB was compared between the mutation groups of the DDR pathways and the DDR wild‐type group utilizing the Kruskal–Wallis test, and the *p* values are shown above the mutation groups. BER, base excision repair; DR, direct reversal of damage; FA, Fanconi anemia; HR, homologous recombination; MMR, mismatch excision repair; NER, nucleotide excision repair; NHEJ, nonhomologous end‐joining; NP, modulation of nucleotide pools; TLS, translesion synthesis; Wild, DDR wild‐type; Mut, DDR mutation; Pathogenic, DDR pathogenic mutation.

A total of 57.8% (26/45) of ESCC cases in the Taizhou dataset had at least one functional impact somatic mutation (Table S2). FA (20.0%) and TLS (20.0%) were the two most frequently mutated pathways, followed by HR (15.6%), MMR (13.3%), and NER (11.1%) (Figure 1A). The most frequently mutated genes were FANCM (6.7%, 3/45), POLE (6.7%, 3/45), HFM1 (4.4%, 2/45), LIG3 (4.4%, 2/45), MLH3 (4.4%, 2/45), POLD1 (4.4%, 2/45), POLG (4.4%, 2/45), POLQ (4.4%, 2/45), and UBE2T (4.4%, 2/45).

In the TCGA dataset, 70.8% (68/96) of cases carried at least one functional impact DDR mutation (Table S2). The most frequently mutated pathway was HR (32.3%), followed by FA (18.8%), TLS (17.7%), and NER (16.6%) (Figure 1B). The most frequently mutated gene was PAXIP1 (8.3%, 8/96), followed by PDSSB (7.3%, 7/96), PRKDC (6.3%, 6/96), BRCA2 (4.2%, 4/96), POLQ (4.2%, 4/96), and REV3L (4.2%, 4/96).

### 3.2 Genomic alterations of DDR genes correlate with TMB and mutational signatures

We compared the TMB between the DDR mutation and DDR wild‐type groups. In both datasets, we observed that the TMB was relatively high in the DDR mutation group. We excluded benign mutations from the DDR mutation group, and the results remained significant (Figure 1C,D). When we separated the DDR mutations into different pathways, in the Taizhou data, the TMB was relatively high in FA, HR, and TLS mutation groups. In the TCGA data, the TMB was relatively high in BER, FA, HR, NER, NHEJ, and TLS mutation groups (Figure S1A,B).

**FIGURE 2:**
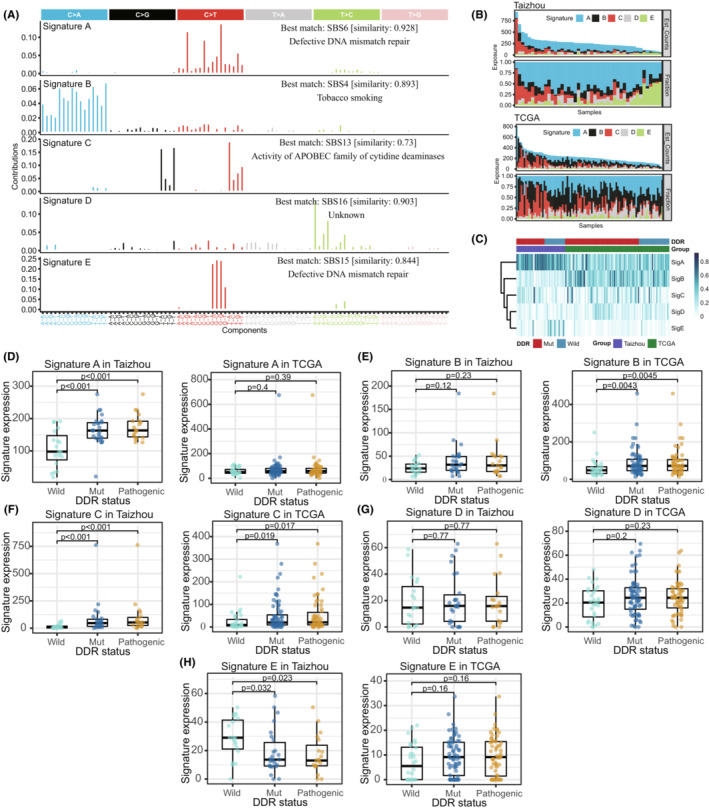
Mutation signature analysis of ESCC. (A) Five de novo signatures were extracted from the Taizhou and TCGA ESCC samples. The matched COSMIC reference signatures and proposed etiology are labeled. (B) The absolute contribution and the relative contribution of the signatures to the mutations of each case in the Taizhou data and TCGA data. (C) The heatmap of signatures in the Taizhou and TCGA samples. (D–H) The expression levels (the number of SNVs) of the signatures in the Taizhou and TCGA data were compared between the DDR mutation groups and the DDR wild‐type group utilizing the Kruskal–Wallis test, and the *p* values are shown above the mutation groups. Wild, DDR wild‐type; Mut, DDR mutation; Pathogenic, DDR pathogenic mutation.

Five de novo signatures (Signature A/B/C/D/E) were extracted from the Taizhou and TCGA ESCC samples and matched to the COSMIC reference signatures (Figure 2A). Signature A, which was similar to COSMIC SBS6 (proposed etiology: MMR deficiency), was the major component of the mutations in most samples. Signature B, which was similar to COSMIC SBS4 (proposed etiology: tobacco smoking), mainly appeared in TCGA samples. Signature C was similar to COSMIC SBS13 (proposed etiology: APOBEC activity). Mutations in some samples mainly consisted of Signature C. Signature D was similar to COSMIC SBS16 (proposed etiology: unknown), and presented in most samples, but the proportion was generally small. Signature E, which was similar to COSMIC SBS15 (proposed etiology: MMR deficiency), mainly appeared in Taizhou samples. (Figure 2B,C)

Furthermore, we analyzed whether DDR mutations contribute to these signatures. In the Taizhou data, the expression of Signature A and Signature C were relatively high in the DDR mutation group, while the expression of Signature E was relatively low in the DDR mutation group (Figure 2D–H). In term of DDR pathways, we observed that Signature A was enriched in FA, HR, and TLS, and Signature C was enriched in BER, FA, HR, and TLS (Figure S2A–E). In the TCGA data, the expression of Signature B and Signature B were relatively high in the DDR mutation group (Figure 2D–H). In term of DDR pathways, we observed that Signature B was enriched in BER, FA, HR, and NER (Figure S2A–E).

### 3.3 Impacts of genomic alterations on the immune microenvironment and patient outcomes

The relative fraction of infiltrating immune cells in all esophageal samples was calculated using the CIBERSORT algorithm with their gene expression data. In both datasets, the infiltration of activated CD4 memory cells, regulatory T cells, and M0/M1 macrophages in the tumor samples increased significantly, while the infiltration of monocytes and resting mast cells was significantly reduced (Figure S3).

We then analyzed the correlation between genomic alterations and the infiltration of immune cells (Figure S4). In the Taizhou data, we found that the eosinophils were enriched in the DDR mutation group. The infiltration of resting dendritic cells was positively correlated with the expression of Signature A and Signature C. In the TCGA data, we found that the TMB was negatively correlated with the infiltration of naïve B cells and activated CD4 memory T cells and was positively correlated with the infiltration of activated mast cells. The expression of Signature A was positively correlated with the infiltration of activated mast cells. The expression of Signature B was negatively correlated with the infiltration of activated CD4 memory T cells and positively correlated with the infiltration of activated mast cells. The expression of Signature C was positively correlated with the infiltration of M0 macrophages and negatively correlated with the infiltration of M2 macrophages.

**FIGURE 3:**
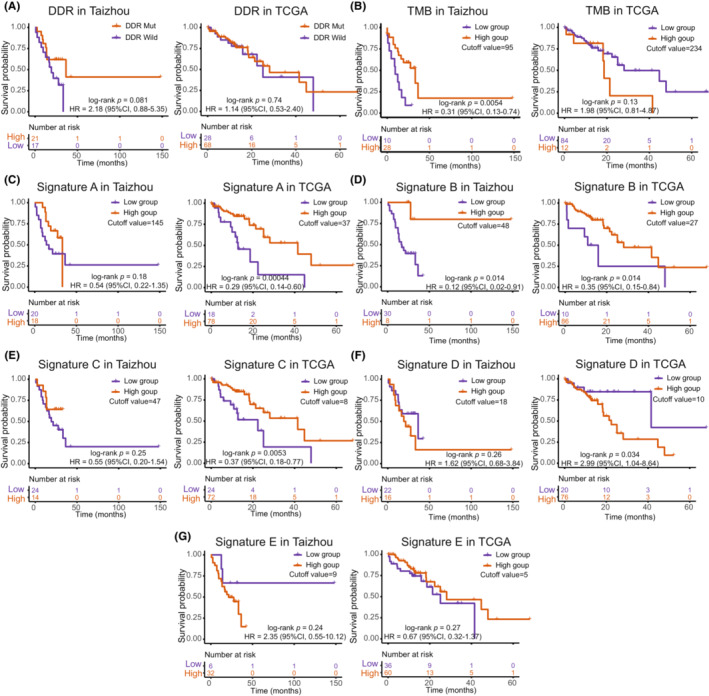
Survival analysis of DDR mutation, tumor mutation burden, and mutational signatures. (A) Kaplan–Meier curves of the DDR mutation and DDR wild‐type groups. (B) Kaplan–Meier curves of high/low tumor mutation burden (TMB) groups. (C–G) Kaplan–Meier curves of high/low expression groups of Signature A (C), Signature B (D), Signature C (E), Signature D (F), Signature E (G). Cutoff values of high/low groups, log‐rank *p* values, hazard ratios (HR), and 95% confidence intervals (CI) are annotated on the graphs.

The OS showed no difference between the DDR mutation and wild groups in both datasets. The OS was significantly different between the high TMB and low TMB groups in the Taizhou data, with the high TMB group exhibiting a good prognosis (Kaplan–Meier analysis, log‐rank *p* = 0.0054, HR = 0.31, 95% CI, 0.13–0.74). The TCGA data showed no difference in OS between the high TMB and low TMB groups. When we analyzed the mutational signatures, the high expression group of Signature B exhibited a good prognosis in the Taizhou data. The high expression groups of Signature A, Signature B, and Signature C exhibited a good prognosis in the TCGA data, while the high expression group of Signature D was associated with a worse prognosis. (Figure 3).

### 3.4 Expression of DDR genes and their relationship with the immune cell infiltration

**FIGURE 4:**
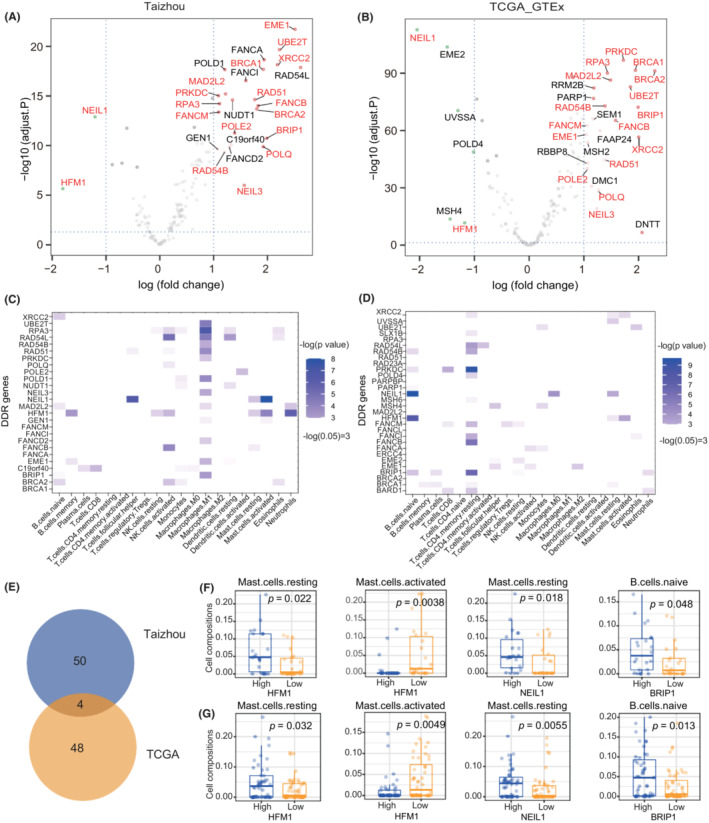
Differentially expressed DNA damage repair (DDR) genes and their correlation with the immune cell infiltration. (A, B) Volcano diagrams of differentially expressed genes (DEGs) of DDR pathways in the Taizhou data (A) and TCGA data (B), where 18 DDR DEGs verified in the two datasets are colored red. (C, D) The significant difference in the immune cell infiltration between the DDR high expression and low expression groups (the median value of the expression was the cutoff value) was evaluated by the Kruskal–Wallis test in the Taizhou data (C) and TCGA data (D). (E) Venn diagram for significant results in the Taizhou data and TCGA data. (F, G) The immune cell infiltration was compared between *HFM1*/*NEIL1*/*BRIP1* high and low expression groups in the Taizhou data (F) and TCGA data (G).

Differential expression analysis was performed between ESCC tumor tissues and paired adjacent normal tissues (Taizhou data) and between TCGA ESCC tumor samples and·GTEx normal esophageal mucosa. A total of 1753 upregulated genes and 2335 downregulated genes were found in Taizhou ESCC samples, and 2820 upregulated genes and 3124 downregulated genes were found in the TCGA ESCC samples. Among them, 30 DEGs were DDR genes in Taizhou samples, and 26 were DDR genes in the TCGA samples. Eighteen DDR genes were differentially expressed in both datasets. Among them, genes in the MMR (*HFM1*) and BER (*NEIL1*) pathways were downregulated in ESCC tumor tissues, while genes in HR (*EME1*, *RAD54L*, *XRCC2*, *BRCA1/2*, *RAD51*, *RAD54B*, *BRIP1*), FA (*UBE2T*, *FANCA*, *FANCI*, *FANCB*, *FANCM*), NER (*RPA3*), NHEJ (*PRKDC*), and TLS (*MAD2L2*) pathways were upregulated in ESCC tumor tissues (Figure 4A,B). Considering the possible influence of DDR mutations on gene expression, we excluded the DDR mutated samples and repeated the analysis, the results were consistent with previous results (17/18 differentially expressed DDR genes were confirmed) except for PRKDC (Figure S5).

The correlation between the relative fractions of several infiltrating immune cells and the DEGs of DDR was evaluated using the Kruskal–Wallis test. The results are shown in Figure 4C–G. In both datasets, the high expression group of *HFM1* had a higher fraction of resting mast cells and a lower fraction of activated mast cells; the high expression group of *NEIL1* had a higher fraction of resting mast cells; and the high expression group of *BRIP1* had a higher fraction of naïve B cells. Furthermore, we combined the two datasets and applied multiple linear regression to evaluate the independent effect of *HFM1*/*NEIL1*/*BRP1* on immune cell infiltration (Table 2). The dataset groups and the TMB were used as covariates. The results showed that the expression of *HFM1* was significantly correlated with the infiltration of resting mast cells (coefficient = 0.026, *p* value = 0.028), and the expression of *NEIL1* was significantly correlated with the infiltration of activated mast cells (coefficient = −0.020, *p* value < 0.001) and resting mast cells (coefficient = 0.012, *p* value = 0.013).

**TABLE 2:** Multivariable model evaluating the independent effect of DDR genes on the infiltration of immune cells.

Gene	Immune cell	Coefficient^a^	*p* ^a^	Coefficient^b^	*p* ^b^
HFM1	Activated mast cell	−0.024	0.051	−0.024	0.052
HFM1	Resting mast cell	0.026	0.026	0.026	0.028
NEIL1	Activated mast cell	−0.019	<0.001	−0.020	<0.001
NEIL1	Resting mast cell	0.012	0.015	0.012	0.013
BRIP1	Naïve B cell	0.001	0.784	0.002	0.746

^a^ The dataset group (Taizhou or TCGA) was used as a covariate in the multiple linear regression model.

^b^ The dataset group and tumor mutation burden were used as covariates in the multivariate linear regression model.

### 3.5 Enrichment analysis and prognostic value of HFM1/NEIL1

**FIGURE 5:**
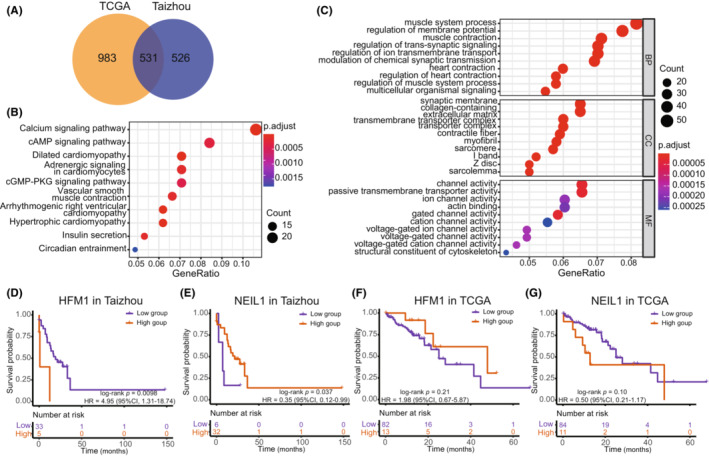
Functional enrichment and survival analyses of DDR genes. (A) Venn diagram of the differentially expressed genes (DEGs) based on comparing the high and low expression of the *HFM1* ESCC samples (the median value of the expression in the normal samples was the cutoff value) in the Taizhou and TCGA data. KEGG (B) and GO (C) analysis results based on the DEGs. (D–G) Kaplan–Meier curves of *HFM1*/*NEIL1* high and low expression groups with overall survival time in the Taizhou data (D, E) and TCGA data (F, G).

We performed differential expression analysis between high‐ and low‐expression groups of *HFM1*/*NEIL1* in tumor samples with the median value as a cutoff in both datasets. And 531 repeated DEGs were confirmed for *HFM1* (Figure 5A), and 11 repeated DEGs were confirmed for *NEIL1*. The DEGs of *HFM1* were subjected to GO and KEGG functional enrichment analyses (Figure 5B,C). The top KEGG pathways identified were the calcium signaling pathway and the cAMP signaling pathway. The DEGs were mainly enriched in biological process (BP) terminology associated with muscle system processes, cellular component (CC) terminology related to the synaptic membrane, and molecular function (MF) terminology associated with channel activity.

The OS was significantly different between the high‐ and low‐expression groups of *HFM1* in the Taizhou data, with the high expression group of *HFM1* exhibiting a worse prognosis (Kaplan–Meier analysis, log‐rank *p* value = 0.0098, HR = 4.95, 95% CI 1.31–18.74). Regarding *NEIL1*, high expression of *NEIL1* was associated with a good prognosis (Kaplan–Meier analysis, log‐rank *p* value = 0.037, HR = 0.35, 95% CI, 0.12–0.99). However, there was no reproducible significant overall survival correlation in the TCGA data. (Figure 5D–G).

## 4 DISCUSSION

The essential roles of DDR in tumorigenesis and antitumor immunity have been discovered in many cancers.^6^, ^13^ However, the effects of DDR on tumor mutation processes and the immune environment in ESCC remain less well explored. In the present study, we investigated genomic and transcriptomic changes in DDR pathways and the profiles of tumor‐infiltrating immune cells in ESCC cases from Taizhou and the TCGA project. We found that mutations in DDR genes commonly occurred in ESCC, contributing significantly to the TMB. Several DDR‐related mutational signatures were identified and associated with immune cell infiltration. In addition, we found that two DDR genes, *HFM1* and *NEIL1*, were suppressed in ESCC and confirmed their significance in the infiltration of mast cells and patient outcomes.

Alterations in DDR pathways play important roles in the development of cancers. Approximately 30% of TCGA PanCanAtlas cancer types have significantly enriched somatic mutations in DDR genes.^7^ The prevalence of somatic mutations in DDR pathways was reported in a Chinese ESCC cohort.^38^ The author defined a gene set of 79 DDR genes encompassing 7 DDR pathways, and found that HR and NHEJ carried significant gene mutations. We focused on 160 DDR genes in nine pathways to detect the genomic alterations of the DDR genes. Consistent with previous studies, our results showed that mutations in DDR commonly occurred in ESCC. We found significant mutations in the HR, FA, and TLS pathways. Furthermore, we observed overexpression of DDR genes in ESCC tumor tissues, including those in the HR and FA pathways. These findings highlight the crucial role that DDR genes play in ESCC development.

Deficiency in DDR can cause genomic aberrations and accelerate the accumulation of mutations in cells, which may lead to carcinogenesis.^6^ The relationship between TMB and DDR genes has been explored in various cancer types, including lung squamous cell carcinoma, urothelial cancer, and gastrointestinal cancer.^9^, ^10^, ^11^ We found that mutations in various DDR pathways were associated with high TMB, as expected. We further found that the mutation of DDR pathways contributed to several mutational signatures in ESCC samples, including the MMR deficiency signature, the APOBEC activity signature, and the smoking‐related signature. Mutational signatures reflect the mutation process of cells caused by endogenous or exogenous carcinogens.^39^ These results suggest that DDR defects may have triggered or prompted multiple mutational processes in ESCC. Nevertheless, additional experimental evidence and data comparing normal and tumor cells are needed to confirm this hypothesis.

DNA damage and genomic instability have been found to shape the antitumor immune response.^13^ A high TMB has been shown to contribute to the generation of immunogenic neopeptides, which are critical for inducing tumor‐specific T‐cell‐mediated immunity after inhibiting checkpoint signals.^40^ Previous studies reported that patients with high TMB might benefit from immunotherapy or targeted therapies.^41^, ^42^ In our study, we found that ESCC patients in the Taizhou data with high TMB tended to have a good outcome. However, this result was not verified in the TCGA data. Interestingly, when we analyzed the mutational signatures, we found that they could predict patient outcomes in the TCGA cohort. In a previous study on a Chinese ESCC cohort, the author reported that mutational signatures could identify patients with different outcomes.^41^ The prognostic value of mutational signatures was also confirmed in other cancers.^43^ We further discovered that the TMB and mutational signatures were correlated with the infiltration of immune cells, including dendritic cells, mast cells, and macrophages. These immune cells are all necessary for T‐cell‐mediated cancer immunity. These findings revealed the impact of genomic instability on the tumor immune environment and potentially explained the prognostic value of the TMB and mutational signatures for the response to immunotherapy. Additionally, it would be beneficial to gain a mechanistic understanding of why specific mutational signatures are associated with different prognoses, for example, whether the response to a specific therapy is influenced by the biological processes underlying a particular mutational signature.

We observed that *HFM1* and *NEIL1* were downregulated in ESCC tumor tissues. Suppression of these two DDR genes suggests they play an essential role in ESCC initiation and progression. We found that the expression levels of *HFM1* and *NEIL1* were associated with patient survival. The prognostic value of *HFM1* and *NEIL1* were previously reported in rectal and prostate cancers, but their effect on immune cell infiltration remains unclear.^44^, ^45^, ^46^ We subsequently found that *HFM1* and *NEIL1* had an independent effect on the infiltration of mast cells after adjusting for TMB. The functional enrichment analysis of the DEGs based on comparing samples with high and low *HFM1* expression indicated that *HFM1* might affect various cancer progression‐related pathways, such as the calcium signaling pathway and the cAMP signaling pathway. *HFM1* and *NEIL1* may serve as potential prognostic biomarkers, but the carcinogenesis of these genes in ESCC needs to be further validated in vivo and in vitro, as does their role in the immune response.

One limitation of our study is that the relationship between the genomic landscape of ESCC and its epidemiological, pathological and other molecular characteristics remains unraveled. As a complement to this study, gathering complete information on ESCC risk factors and sequencing approaches that determine the epigenome and proteome will be an important next step in understanding the molecular changes that DDR defects cause and their mechanisms. In addition, the hypotheses generated from this study will require experimental and clinical validation. The impact of mutations on immune responses needs to be confirmed in biological experiments, and the candidate predictive biomarkers identified in this study need to be evaluated in clinical trials.

In conclusion, our study identified a ubiquitous deficiency of DDR in ESCC and showed its essential role in tumor mutational processes. We further confirmed the association between genomic mutations caused by DDR deficiency and tumor‐infiltrating immune cells. We found that *HFM1* and *NEIL1* were suppressed in ESCC and were associated with immune cell populations and patient survival. These findings reinforce the importance of DDR gene function in cancer development and the immune response in ESCC.

## AUTHOR CONTRIBUTIONS

**Huangbo Yuan:** Conceptualization (lead); methodology (lead); resources (equal); software (equal); visualization (lead); writing – original draft (lead); writing – review and editing (lead). **Tao Qing:** Conceptualization (equal); methodology (equal); writing – review and editing (equal). **Sibo Zhu:** Methodology (equal); supervision (equal); writing – review and editing (equal). **Xiaorong Yang:** Investigation (equal); resources (equal); writing – review and editing (equal). **Weicheng Wu:** Methodology (equal); resources (equal); writing – review and editing (equal). **Kelin Xu:** Conceptualization (equal); writing – review and editing (equal). **Hui Chen:** Investigation (equal); writing – review and editing (equal). **Yanfeng Jiang:** Investigation (equal); resources (equal); writing – review and editing (equal). **Chengkai Zhu:** Software (equal); visualization (equal); writing – review and editing (equal). **Ziyu Yuan:** Investigation (equal); resources (equal); writing – review and editing (equal). **Tiejun Zhang:** Supervision (equal); writing – review and editing (equal). **Li Jin:** Project administration (equal); supervision (equal); writing – review and editing (equal). **Chen Suo:** Conceptualization (equal); supervision (equal); writing – review and editing (equal). **Ming Lu:** Investigation (equal); supervision (equal); writing – review and editing (equal). **Xingdong Chen:** Conceptualization (equal); funding acquisition (equal); project administration (equal); supervision (equal); writing – review and editing (equal). **Weimin Ye:** Conceptualization (equal); methodology (equal); writing – review and editing (equal).

## FUNDING INFORMATION

This work was supported by the National Natural Science Foundation of China (grant numbers: 82073637, 81973116, 91846302), the National Key Research and Development Program of China (grant number: 2019YFC1315804, 2017YFC0907000), the Innovation Grant from Science and Technology Commission of Shanghai Municipality, China (grant number: 20ZR1405600), three‐Year Action Plan for Strengthening Public Health System in Shanghai (grant number: GWV‐10.2‐YQ32).

## CONFLICT OF INTEREST STATEMENT

The authors declare that they have no known competing financial interests or personal relationships that could have appeared to influence the work reported in this paper.

## ETHICAL APPROVAL STATEMENT

The protocols for sample collection were approved by the Institutional Ethics Committee of the School of Life Sciences, Fudan University, and Qilu Hospital of Shandong University. All participants or their proxies provided informed consent.

## Supporting information


Figure S1

Figure S2

Figure S3

Figure S4

Figure S5

Table S1

Table S2

## Data Availability

The exome sequencing and RNA sequencing data of 45 Chinese ESCC samples have been deposited in the National Omics Data Encyclopedia of China (NODE, http://www.biosino.org/node/index) under Bioproject (accession number: OEP000143, http://www.biosino.org/node/project/detail/OEP000143). All relevant data sets are available upon request from the corresponding author.
