# Supplementary material for: The effects of altered DNA damage repair genes on mutational processes and immune cell infiltration in esophageal squamous cell carcinoma
Source: Cancer Med. 2023 Jan 27;12(8):10077–90. doi: 10.1002/cam4.5663 (PMC10166979; doi:10.1002/cam4.5663)
Supplement: Supplementary file 1 — Figure S1 [file CAM4-12-10077-s003.pdf]

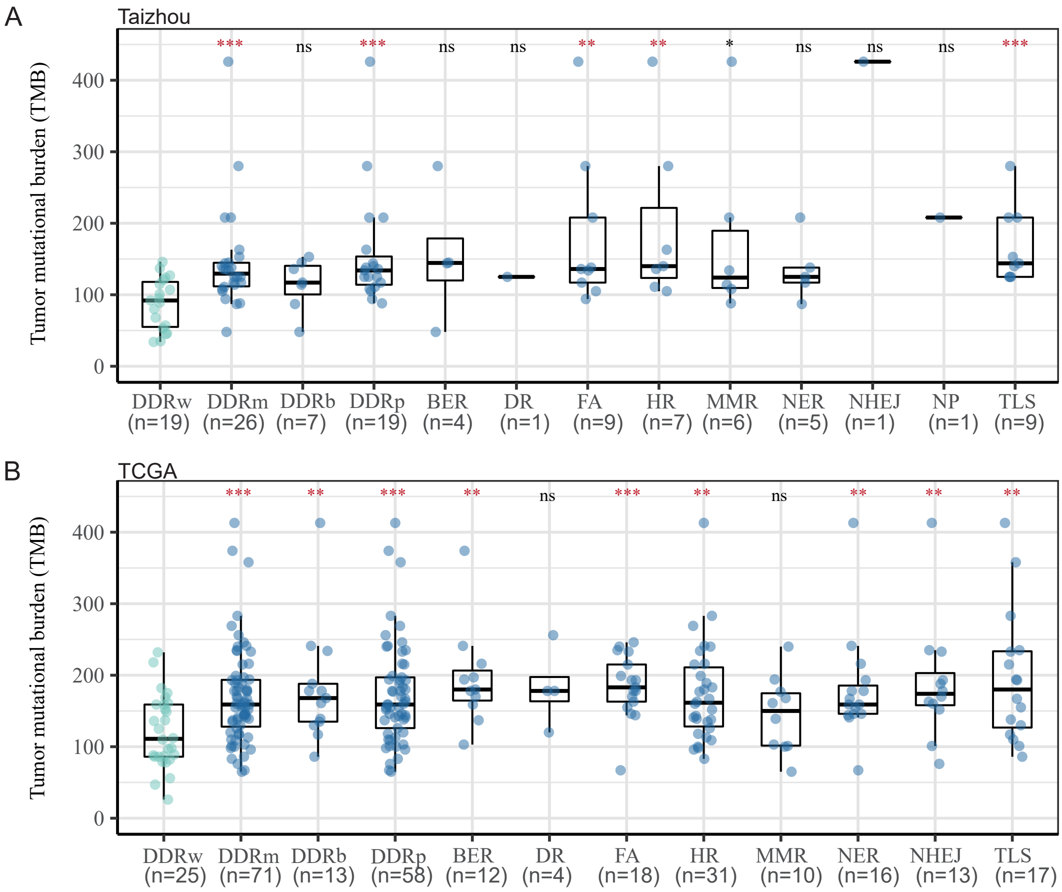

Figure S1. Tumor mutation burdens (TMB) of DDR pathways in the Taizhou data (A) and TCGA data (B). TMB was compared between the mutation groups of the DDR pathways and the DDR wild-type group utilizing the Kruskal–Wallis test, and the p values are shown above the mutation groups. . Abbreviations: DDRw, DDR wild-type; DDRm, DDR mutation; DDRb, DDR benign mutation;DDRp, DDR pathogenic mutation. BER, base excision repair; DR, direct reversal of damage; FA, Fanconi anemia; HR, homologous recombination; MMR, mismatch excision repair; NER, nucleotide excision repair; NHEJ, nonhomologous end-joining; NP, modulation of nucleotide pools; TLS, translesion synthesis. *, $p < 0.05$; **, $p < 0.01$; ***, $p < 0.001$; ns, nonsignificant. Results that remain significant after FDR correction are hightlighted in red.
