# Supplementary material for: The effects of altered DNA damage repair genes on mutational processes and immune cell infiltration in esophageal squamous cell carcinoma
Source: Cancer Med. 2023 Jan 27;12(8):10077–90. doi: 10.1002/cam4.5663 (PMC10166979; doi:10.1002/cam4.5663)
Supplement: Supplementary file 2 — Figure S2 [file CAM4-12-10077-s007.pdf]

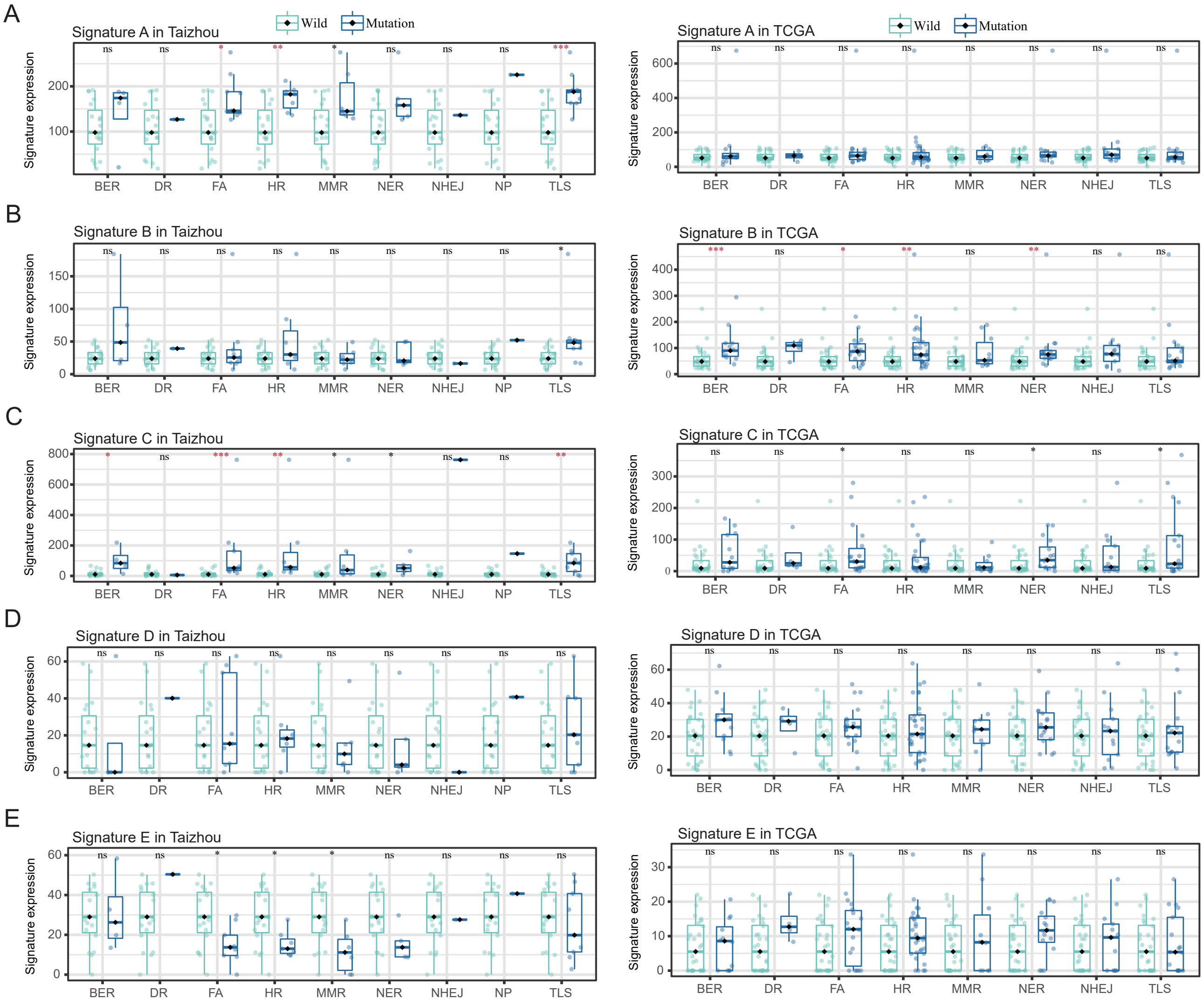

Figure S2. Expression of signatures of DDR pathways in the Taizhou data (A) and TCGA data (B).
The expression levels of Signature A (A), Signature B (B), Signature C (C), Signature D (D), Signature E (E) were compared between the mutation groups of the DDR pathways and the DDR wild-type group utilizing the Kruskal–Wallis test, and the p values are shown above the mutation groups. Abbreviations: BER, base excision repair; DR, direct reversal of damage; FA, Fanconi anemia; HR, homologous recombination; MMR, mismatch excision repair; NER, nucleotide excision repair; NHEJ, nonhomologous end-joining; NP, modulation of nucleotide pools; TLS, translesion synthesis. *, $p < 0.05$; **, $p < 0.01$; ***, $p < 0.001$; ns, nonsignificant. Results that remain significant after FDR correction are hightlighted in red.
