# Supplementary figures and images for: The effects of altered DNA damage repair genes on mutational processes and immune cell infiltration in esophageal squamous cell carcinoma
Source: Cancer Med. 2023 Jan 27;12(8):10077–90. doi: 10.1002/cam4.5663 (PMC10166979; doi:10.1002/cam4.5663)

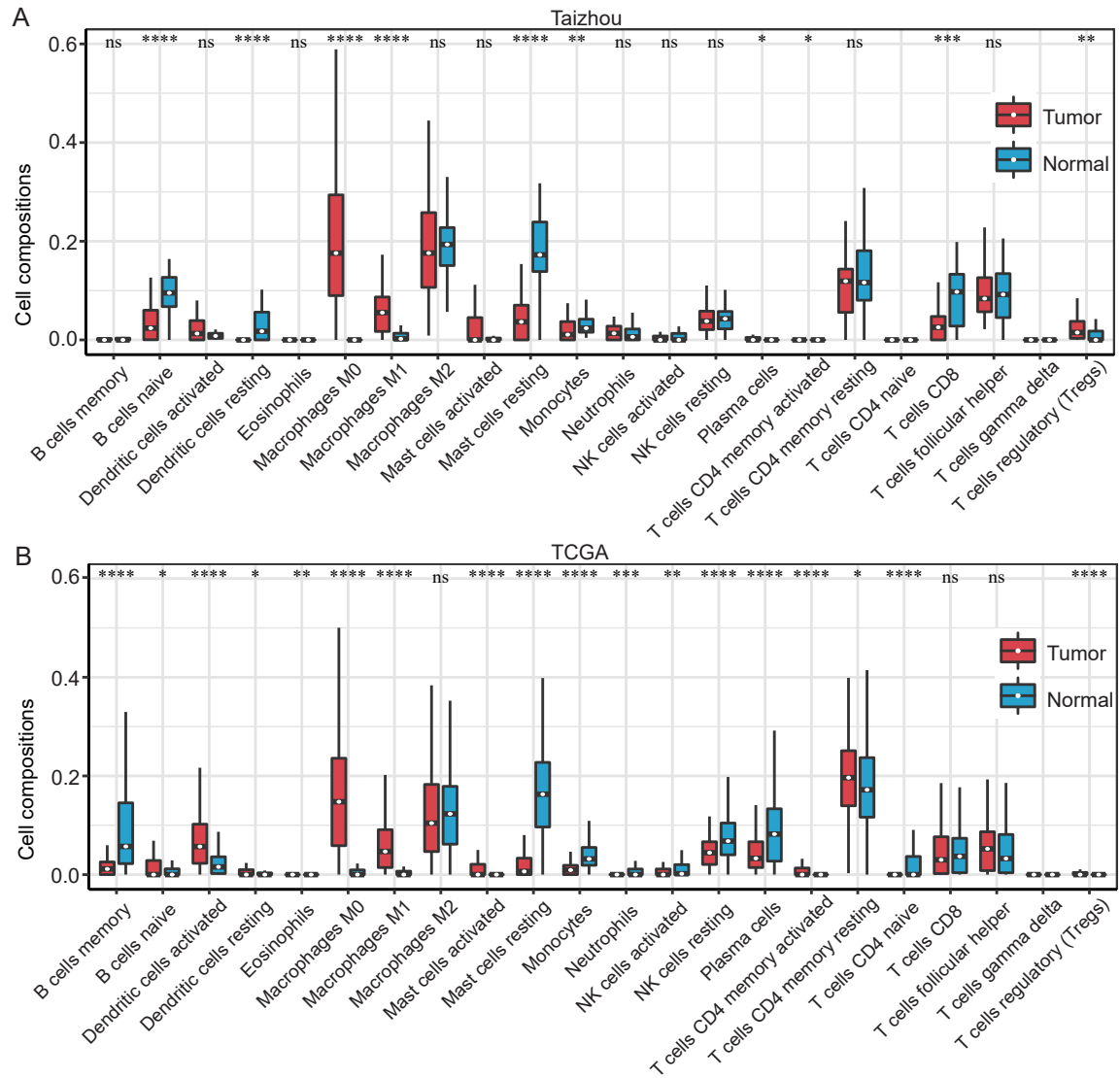

Figure S3. Profiles of immune cell infiltration in Taizhou (A) and TCGA (B) ESCC cases

Supplement: Supplementary file 3 — Figure S3 [file CAM4-12-10077-s001.pdf]
