# Supplementary material for: The effects of altered DNA damage repair genes on mutational processes and immune cell infiltration in esophageal squamous cell carcinoma
Source: Cancer Med. 2023 Jan 27;12(8):10077–90. doi: 10.1002/cam4.5663 (PMC10166979; doi:10.1002/cam4.5663)
Supplement: Supplementary file 4 — Figure S4 [file CAM4-12-10077-s002.pdf]

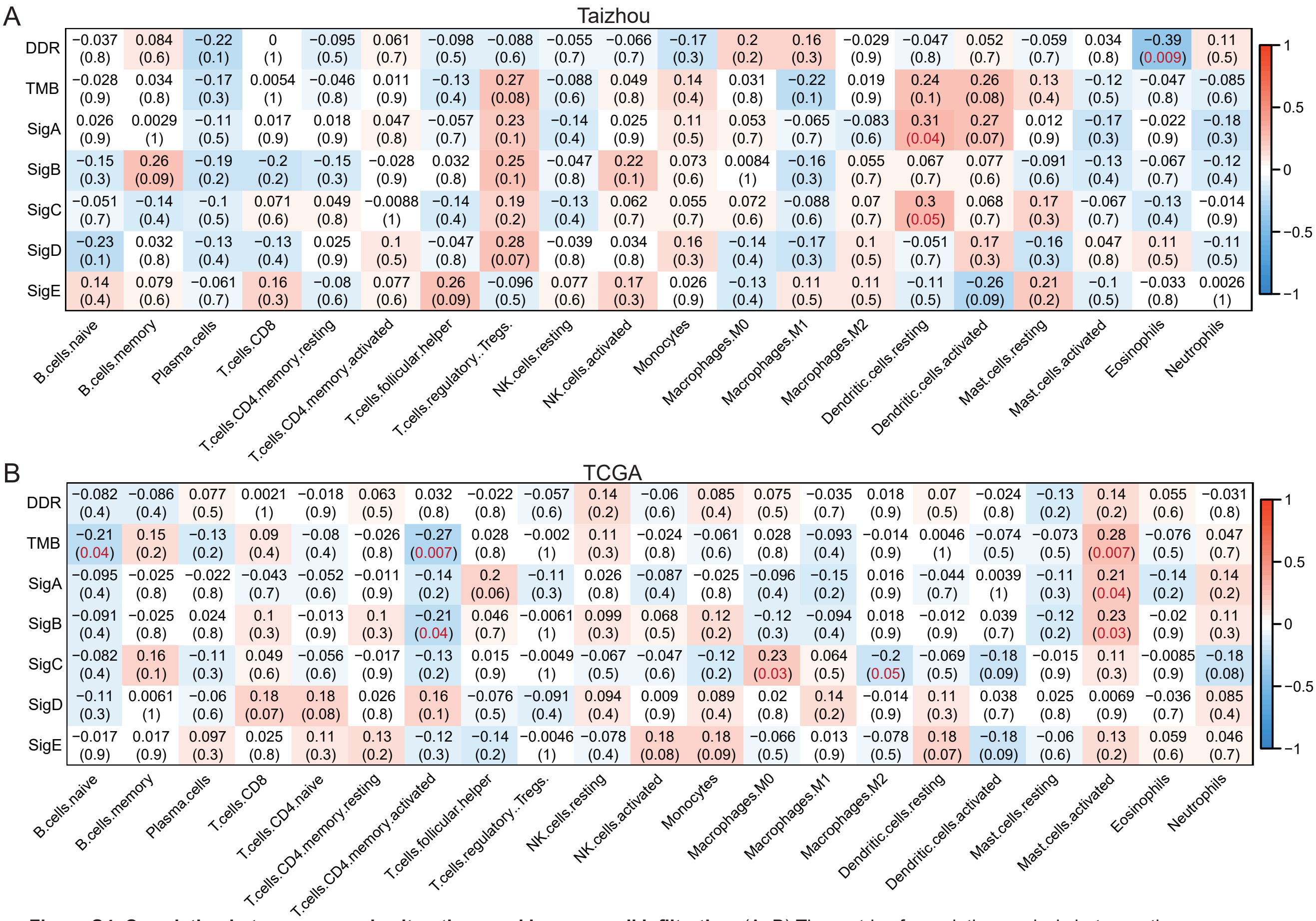

Figure S4. Correlation between genomic alterations and immune cell infiltration. (A, B) The matrix of correlation analysis between the DDR mutation, tumor mutation burden (TMB), expression of mutational signatures, and the immune cell infiltration in the Taizhou data (A) and TCGA data (B). Pearson's correlation coefficients and *p* values are labeled in the grids, and significant results are highlighted in red.
