# Supplementary material for: The effects of altered DNA damage repair genes on mutational processes and immune cell infiltration in esophageal squamous cell carcinoma
Source: Cancer Med. 2023 Jan 27;12(8):10077–90. doi: 10.1002/cam4.5663 (PMC10166979; doi:10.1002/cam4.5663)
Supplement: Supplementary file 5 — Figure S5 [file CAM4-12-10077-s005.pdf]

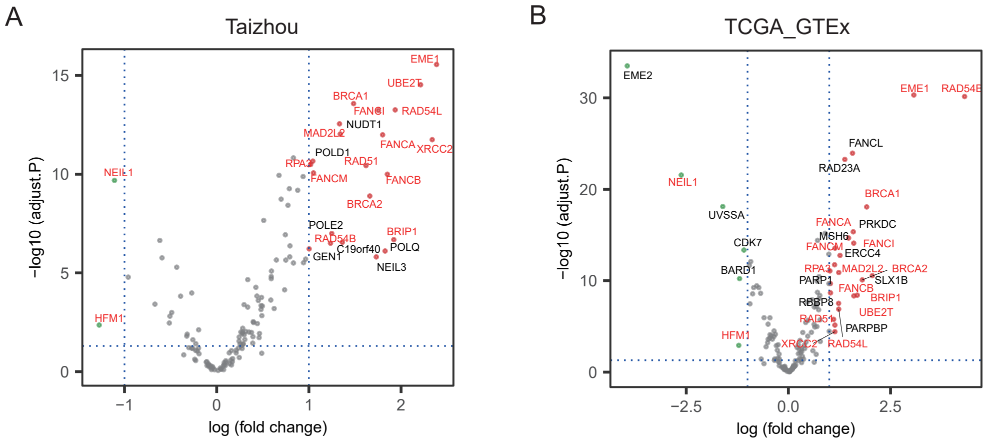

Figure S5. Differentially expressed DNA damage repair (DDR) genes after excluding DDR mutation samples. Volcano diagrams of differentially expressed genes (DEGs) of DDR pathways in the Taizhou data (A) and TCGA data (B), where 17 DDR DEGs verified in the two datasets are colored red.
