# Supplementary material for: The effects of altered DNA damage repair genes on mutational processes and immune cell infiltration in esophageal squamous cell carcinoma
Source: Cancer Med. 2023 Jan 27;12(8):10077–90. doi: 10.1002/cam4.5663 (PMC10166979; doi:10.1002/cam4.5663)
Supplement: Supplementary file 6 — Table S1 [file CAM4-12-10077-s006.docx]

**Table S1: Summary of DNA damage repair genes**

| Pathway | Gene name (synonyms) | Number of genes |
| --- | --- | --- |
| Base excision repair (BER) | UNG, SMUG1, MBD4, TDG, OGG1, MUTYH (MYH), NTHL1 (NTH1), MPG, NEIL1, NEIL2, NEIL3, APEX1 (APE1), APEX2, LIG3, XRCC1, PNKP, APLF, HMCES, PARP1 (ADPRT), PARP2 (ADPRTL2), PARP3 (ADPRTL3), PARG, PARPBP | 23 |
| Direct reversal of damage (DR) | MGMT, ALKBH2 (ABH2), ALKBH3 (DEPC1) | 3 |
| Mismatch repair (MMR) | MSH2, MSH3, MSH6, MLH1, PMS2, MSH4, MSH5, MLH3, PMS1, PMS2P3 (PMS2L3), HFM1 | 11 |
| Nucleotide excision repair (NER) | XPC, RAD23B, CETN2, RAD23A, XPA, DDB1, DDB2 (XPE), RPA1, RPA2, RPA3, TFIIH, ERCC3 (XPB), ERCC2 (XPD), GTF2H1, GTF2H2, GTF2H3, GTF2H4, GTF2H5 (TTDA), GTF2E2, CDK7, CCNH, MNAT1, ERCC5 (XPG), ERCC1, ERCC4 (XPF), LIG1, NER-related, ERCC8 (CSA), ERCC6 (CSB), UVSSA (KIAA1530), XAB2 (HCNP), MMS19 | 32 |
| Homologous recombination (HR) | RAD51, RAD51B, RAD51D, HELQ (HEL308), SWI5, SWSAP1, ZSWIM7 (SWS1), SPIDR, PDS5B, DMC1, XRCC2, XRCC3, RAD52, RAD54L, RAD54B, BRCA1, BARD1, ABRAXAS1, PAXIP1 (PTIP), SMC5, SMC6, SHLD1, SHLD2 (FAM35A), SHLD3, SEM1 (SHFM1) (DSS1), RAD50, MRE11A, NBN (NBS1), RBBP8 (CtIP), MUS81, EME1 (MMS4L), EME2, SLX1A (GIYD1), SLX1B (GIYD2), GEN1 | 35 |
| Fanconi anemia (FA) | FANCA, FANCB, FANCC, BRCA2 (FANCD1), FANCD2, FANCE, FANCF, FANCG (XRCC9), FANCI (KIAA1794), BRIP1 (FANCJ), FANCL, FANCM, PALB2 (FANCN), RAD51C (FANCO), SLX4(FANCP), FAAP20 (C1orf86), FAAP24 (C19orf40), FAAP100, UBE2T (FANCT) | 19 |
| Non-homologous end joining (NHEJ) | XRCC6 (Ku70), XRCC5 (Ku80), PRKDC, LIG4, XRCC4, DCLRE1C (Artemis), NHEJ1 (XLF, Cernunnos) | 7 |
| Modulation of nucleotide pools (NP) | NUDT1 (MTH1), DUT, RRM2B (p53R2), PARK7 (DJ-1), DNPH1, NUDT15 (MTH2), NUDT18 (MTH3) | 7 |
| Translesion DNA synthesis (TLS) | POLA1, POLB, POLD1, POLD2, POLD3, POLD4, POLE (POLE1), POLE2, POLE3, POLE4, REV3L (POLZ), MAD2L2 (REV7), REV1 (REV1L), POLG, POLH, POLI (RAD30B), POLQ, POLK (DINB1), POLL, POLM, POLN (POL4P), PRIMPOL, DNTT | 23 |
